# An Organofluorine Isoselenocyanate Analogue of Sulforaphane Affects Antimetabolite 5-Fluorouracil’s Anticancer Activity: A Perspective for New Combinatory Therapy in Triple-Negative Breast Cancer

**DOI:** 10.3390/molecules28155808

**Published:** 2023-08-01

**Authors:** Małgorzata Milczarek, Tomasz Cierpiał, Piotr Kiełbasiński, Milena Małecka-Giełdowska, Marta Świtalska, Joanna Wietrzyk, Maciej Mazur, Katarzyna Wiktorska

**Affiliations:** 1 Laboratory of Translation Research, Department of Biomedical Research, National Medicines Institute, Chełmska 30/34, 00-725 Warsaw, Poland; 2 Division of Organic Chemistry, Centre of Molecular and Macromolecular Studies, Polish Academy of Sciences, Sienkiewicza 112, 90-363 Łódź, Poland; tomasz.cierpial@cbmm.lodz.pl (T.C.); piotr.kielbasinski@cbmm.lodz.pl (P.K.); 3 Department of Laboratory Medicine, Medical University of Warsaw, Stefana Banacha 1A, 02-097 Warsaw, Poland; 4 Central Laboratory, Central Teaching Hospital University Clinical Center, Medical University of Warsaw, Stefana Banacha 1A, 02-097 Warsaw, Poland; 5 Hirszfeld Institute of Immunology and Experimental Therapy, Polish Academy of Sciences, Rudolfa Weigla 12, 53-114 Wrocław, Poland; marta.switalska@hirszfeld.pl (M.Ś.); joanna.wietrzyk@hirszfeld.pl (J.W.); 6 Faculty of Chemistry, University of Warsaw, Ludwika Pasteura 1, 02-093 Warsaw, Poland; mmazur@chem.uw.edu.pl; 7 Department of Physics and Biophysics, Institute of Biology, Warsaw University of Life Sciences—SGGW, Nowoursynowska 166, 02-776 Warsaw, Poland

**Keywords:** antimetabolites, isoselenocyanate, 5-fluorouracil, sulforaphane, anticancer effect

## Abstract

Antimetabolites, especially 5-fluorouracil, are commonly used clinically to treat breast, colon, and other cancers. However, their side effects and inefficiency in monotherapy have prompted further searches for new combinations. Thus, the anticancer effect of 5-fluorouracil (5-FU) and the sulforaphane analogue, 4-isoselenocyanato-1-butyl 4′-fluorobenzyl sulfoxide (ISC), were tested in in vitro and in vivo models of triple-negative breast cancer (TNBC) as a new option for this treatment-resistant and aggressive type of breast cancer. A synergic interaction between 5-FU and ISC was observed in the TNBC in vitro model MDA-MB-231 cell line, which led to enhanced antiproliferative effects. The results of in vitro studies were confirmed by in vivo tests, which demonstrated stronger tumor growth inhibition and additive interactions between 5-FU and ISC in the murine TNBC model. Moreover, the results of the body mass and blood analysis showed the safety of the tested combination. The mechanistic study revealed that the combined treatment triggered apoptosis and necrosis, as well as inhibited cell migration.

## 1. Introduction

**Figure 1:**
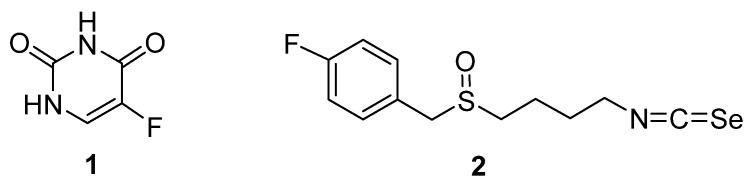
Structural formulas of 5-fluorouracyl 1 and 4-isoselenocyanato-1-butyl 4′-fluorobenzyl sulfoxide 2.

The antimetabolite 5-fluorouracil(5-FU) is a fluoropyrimidine analog (Figure 1). It is a chemotherapy drug used for the treatment of cancers such as colon, head and neck, and breast cancer, including triple-negative breast cancer (TNBC) that is aggressive, therapy resistant, and lacking specific drug targets. TNBC is widely known to be a high metabolic group, and antimetabolic drugs (e.g., 5-FU) are regarded as effective in inhibiting TNBC cell growth [1].

Eighty percent of the 5-FU dose is mainly metabolized in the liver into an inactive form. Approximately 3% of the original dose of 5-FU is metabolized to active metabolites: 5-fluorodeoxyuridine monophosphate (5-FdUMP), 5-fluorodeoxyuridine triphosphate (5-FdUTP), and 5-fluorouridine triphosphate (5-FUTP), which elicit both clinical and toxic effects. 5-FdUMP and 5-FdUTP damage DNA, while 5-FdUMP forms a temporary complex with thymidylate synthase (TS) and leads to deoxynucleotide imbalances and an increase in levels of deoxyuridine triphosphate. 5-FdUTP is incorporated into DNA instead of deoxythymidine triphosphate. 5-FUTP incorporates into cytoplasmic or nuclear RNA and hampers RNA and protein synthesis, which leads to cell death [2,3].

5-FU is commonly administered in combination with other classic anticancer drugs (e.g., platinum-based drugs, taxanes) [4]. Due to insufficient efficacy and the development of multidrug resistance to 5-FU or its combinations, new therapies are being developed. Although targeting nucleotide metabolism is the main goal of many research groups, other ideas have also been developed, e.g., 5-FU was combined with bevacizumab or cetuximab in metastatic colorectal cancer, which exploited the potential effectiveness of the nucleotide analog in enhancing immunotherapy. Interestingly, 5-FU also influences the immune system cells’ response by selectively killing myeloid-derived suppressor cells, and by enhancing T cell-dependent antitumor immunity [5].

In order to enhance the efficacy and reduce the toxicity of 5-FU, combinations of this drug with natural compounds such as curcumin, genistein, Manuka honey, and sulforaphane have been tested [6,7,8]. As was shown in in vivo models, natural compounds exhibit favorable properties and, used in combination with anticancer drugs, synergistically induce cancer cell death. Further, by altering antioxidant enzymes, they also attenuate a toxic effect in normal cells, e.g., sulforaphane was shown to hamper the cardiotoxicity of doxorubicin [9]. Our earlier in vitro study showed that sulforaphane is an effective modulator of 5-FU activity. It acts synergistically with 5-FU in breast and colon cancer cells [10,11]. Moreover, the antagonistic type of interaction between compounds was revealed in normal cells [12].

To find safe and more effective anticancer agents, we tested the organofluorine isoselenocyanate analogs of sulforaphane and among which 4-isoselenocyanato-1-butyl 4′-fluorobenzyl sulfoxide (ISC) 2 (Figure 1) exhibited the most beneficial properties. It bears the 4-fluorobenzyl substituent bonded to the sulfinyl sulfur atom, which is connected with the isoselenocyanate moiety (in place of the isothiocyanate group present in the original sulforaphane), via an alkyl chain, consisting of four methylene groups. We found ISC to exert a significantly higher cytotoxic effect against the breast cancer cell lines than the original sulforaphane, while being less toxic for the human nonmalignant model of a normal cell line [13]. Based on the promising sulforaphane influence on 5-FU activity and ISC encouraging anticancer ability, its combined effects with 5-FU were investigated in in vitro and in vivo TNBC models.

We showed the synergic anticancer effect of 5-FU and ISC combined treatment in in vitro model. Combined treatment induced apoptosis and blocked cell migration. The in vivo study confirmed a beneficial interaction between compounds that led to tumor growth inhibition and showed the systemic safety of the studied combination.

## 2. Results

### 2.1. In Vitro Model

#### Cell Growth and Type of Interaction

The changes in cell growth after combined treatment and after alone treatment were examined using an MTT assay. The studies were conducted on MDA-MB-231cells, which is the most commonly used cell line to study triple-negative breast cancer [14].

**Figure 2:**
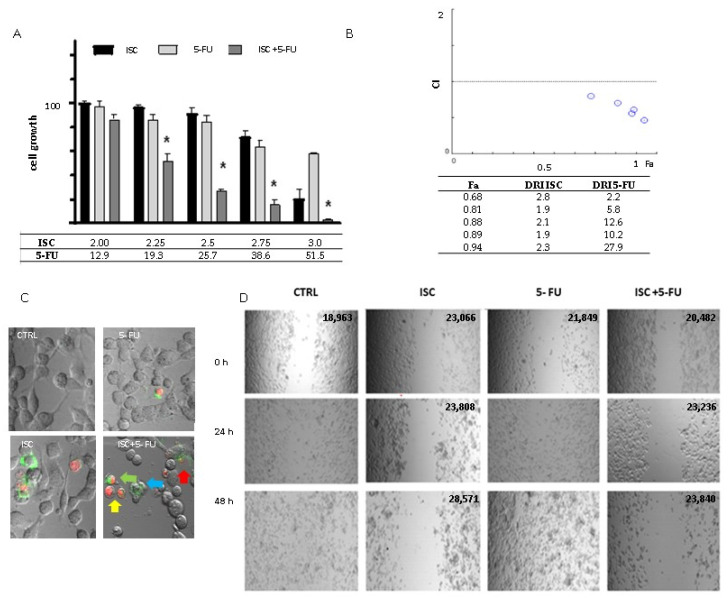
(**A**) Cell growth changes after 5-FU and ISC alone and combined treatments; * Tukey’s test vs. alone administrations of ISC and 5-FU, *p* < 0.05 (**B**) values of CI and DRI with respect to fa; (**C**) apoptosis detection after ISC and 5-FU alone and their combined treatment (apoptosis/necrosis detection after combined treatment, yellow arrow—necrotic cell, blue arrow—early apoptotic cell, green arrow—late apoptotic cell, red arrow—apoptotic body; green—FITC-stained phosphatotydyloserine (hallmark of early apoptosis), red—PI stained cell nuclei of late apoptotic/necrotic cells (**D**) cell migration in control cells (CTRL), ISC alone treatment, 5-FU alone treatment and after combined treatment (ISC + 5-FU). The wound areas (μm^2^) are presented in the upper right corner of each picture.

As shown in Figure 2A, the combined treatment’s effects on the cells were stronger than the effects of both compounds used alone. The most significant differences between combined and alone treatments were observed when 19.3 μM and higher 5-FU concentrations were used in the combined treatment. Combined treatments at the three highest concentrations exhibited at least a 70% decrease in cell number, and those effects were about 4 to 5 times stronger than the effect of the alone treatments (Figure 2A).

On the basis of the MTT results, the types of interaction were defined using the Chou–Talalay method (Figure 2B). The type of interaction was defined in relation to fa (fa—fraction of the cells affected). The value of fa indicates the drop in cell growth, e.g., when cell growth decreased by 70%, the fa is equal to 0.7. The type of interaction was determined based on the Combination Index value: synergism (CI < 0.9), antagonism (CI > 1.1), and an additive effect (CI = 0.9–1.1). In tested cases, values of CI were typical for synergism and decreased when the value of fa increased. At a fa of 0.7, the CI was equal to 0.8, whereas at fa = 0.9, the CI was equal to 0.5.

The Chou–Talalay method allows the dose-reduction index (DRI) value to be determined. The DRI determines by how many folds the concentration of compounds administered in synergic combination may be reduced, compared to alone administration at a given effect level. In the case of all tested combined treatments, DRIs obtained for ISC were near 2. Interestingly, the DRI of 5-FU increased dependently on fa. At a fa of 0.68, the DRI was 10.2, while for a fa of 0.97, the DRI value was 27.9.

At the next stage of study, to characterize the cytotoxic effect of the combined treatment, the apoptosis/necrosis detection experiment was conducted using Annexin V-FITC/PI staining. The early apoptotic cells are stained with AnnexinV-FITC because the plasma membrane loses its asymmetry, and phosphatidylserine translocates from the inner left to the outer one. Late apoptotic or necrotic cells are stained with AnnexinV-FITC and PI due to the high permeability of damaged plasma membrane.

The study was performed after 24 h of incubation. The most intensive changes were observed after 24 h of sequential treatment (Figure 2C). Cells exhibiting the hallmarks of an early stage (green staining membrane) and the late stage of apoptosis (green staining and red staining) were detected in the cell culture. Moreover, apoptosis detection was performed via observation of apoptosis bodies. In addition, we also noticed necrotic cells (only red staining cells, Figure 2C). Necrotic cells were mainly detected after combined treatment. After 5-FU alone treatment, the least changes were observed (a only few apoptotic cells), while after ISC alone treatments, the apoptotic and necrotic cells were noticed (Figure 2C).

Because TNBC is a highly metastatic type of cancer, the effect of combination treatment on cell migration was also investigated with a wound healing assay. As shown in Figure 2D, after the combined treatment as well as alone ISC treatment, the motility of the MDA-MB-231 cells was the most significantly inhibited. In those cases, the wound areas were similar. In the control cells and cells treated with 5-FU alone, the wound’s closure was practically complete after 24 h, and there was no evidence of the wound after 48 h.

### 2.2. In Vivo Studies

#### 2.2.1. Tumor Growth and Metastasis in Lung

The observed beneficial properties of combining 5-FU with ISC in in vitro conditions encouraged us to perform studies in the mammary gland carcinoma 4T1 (TNBC) animal model. The 4T1 cells were inoculated orthotopically in to BALB/c mice. When the tumors were palpable, mice were randomly divided into uniform groups and the compounds administration was performed (ISC at the dose of 50 mg/kg i.p., and 5-FU at the dose of 100 mg/kg i.v.). Taking into account the immunomodulatory properties of sulforaphane, a syngeneic model where mice have a fully functional immune system was chosen. In the case of xenografting human cells, we would have to use a mouse model with impaired immune function and thus omit one of the potential mechanisms of sulforaphane activity.

The anticancer activity of the studied compounds and their combination was evaluated based on tumor volume change and the number of metastases counted.

**Figure 3:**
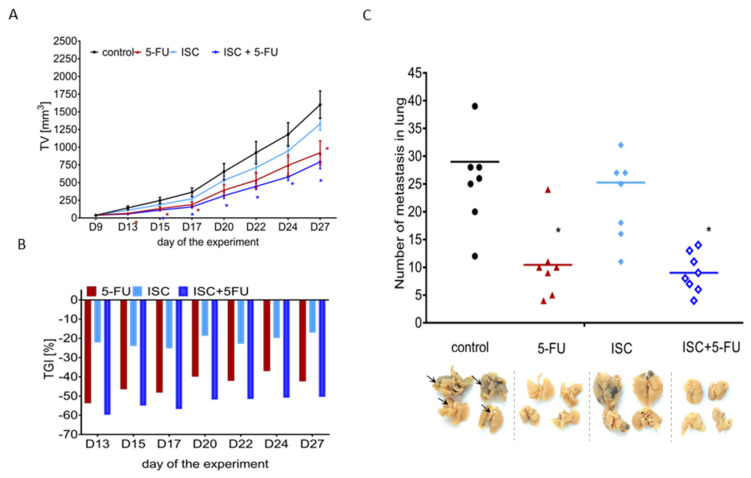
(**A**) Tumor growth kinetics (mean of tumor volume (TV)); (**B**) tumor growth inhibition (TGI) after 5-FU and ISC alone treatments and after their combination treatments; (**C**) number of metastases in the lung after 5-FU and ISC alone treatments and after their combination treatments, images of four representative formalin−fixed lungs, arrows point at metastatic foci in control sample. * Mann–Whitney U test vs. control; *p* < 0.05.

As shown in Figure 3A, tumor growth kinetics (mean of tumor volume (TV) significantly decreased after 5-FU alone treatment and after combined treatment in comparison to the control group. After 27 days of the experiment, TV after combined treatment was 2 times smaller in comparison to the untreated group. Tumor growth inhibition (TGI) observed after 5-FU alone treatment was at a level near 40–50%. The value of TGI after combined treatment was near 60%, whereas ISC alone treatments reduced tumor volume by 20% (Figure 3B). Based on the experimental TGI and the value of the hypothetical TGI, the type of interaction between the tested compounds was determined. The quantitative analysis of the interaction type between ISC and 5-FU showed a primarily additive effect. In turn, after 24 days, synergism was noticed and the hypothetical TGI values were lower than the value of TGI calculated based on the experimental results (Table 1).

At the end of the experiment (Day 27), the metastatic activity was tested. Animals were sedated for blood collection and then sacrificed for lung collection. The number of metastases was counted in lungs fixed with 5% (*v*/*v*) paraformaldehyde in PBS. All tested treatments decreased the number of metastases in the lung in comparison to the control group. After the 5-FU alone treatment and after combined treatment, similar effects were observed, and the number of metastases in the lung dropped by 50% in comparison to the control (Figure 3C).

#### 2.2.2. Biochemical Tests and Blood Count

**Figure 4:**
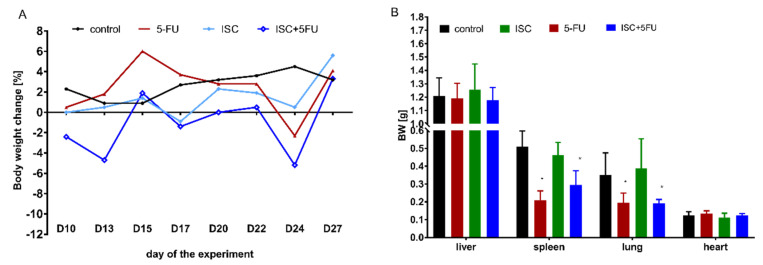
(**A**) Body weight change, (**B**) organ weight after 5−FU and ISC alone, and after their combination treatments. * Mann–Whitney U test vs. the control; *p* < 0.05.

To determine the safety of the tested compounds, blood morphology (Table 2), biochemical parameters of blood (Table 3), and total body and organs’ (liver, spleen, heart, and lung) weight change (Figure 4) were examined.

Blood count tests showed significant differences after treatment with 5-FU alone and after combination treatment compared to untreated mice bearing TNBC (Table 2). The exception was the platelet count study. An increase in platelet counts was observed in mice receiving the combination treatment. The level was three times higher than in healthy mice and significantly different from treatment with ISC and 5-FU alone. In the case of TNBC-bearing mice, the level of leukocytes was elevated (this is observed in mice bearing 4T1 cells), and the ratio of the level of lymphocytes to the level of granulocytes was altered in comparison to healthy mice. The treatment with 5-FU alone as well as combined treatment with 5-FU and ISC inhibited leukocytosis and fully recovered normal lymphocytes-granulocytes ratio. In groups receiving 5-FU (alone and in combination), a decreased number of erythrocytes and a decreased level of hemoglobin and hematocrit were observed in comparison to the control mice, as well as in the group treated with ISC + 5-FU, also in comparison to healthy mice.

To assess the function of the liver, kidney, and heart, biochemical tests were conducted (Table 3). Combined treatment enhanced 5-FU’s capability to lower the level of hepatic enzyme AST (aspartate aminotransferase) and ALT (alanine aminotransferase) compared to control mice. The level of the myocardial CK-MB isoform was also lowest in the group receiving ISC with 5-FU. In the case of creatinine, CK, and urea, no significant changes after 5-FU, ISC, and their combination treatments were observed. The analysis of CK showed very large differences between the mice in each group.

The tested types of treatments showed no toxic effects in mice, as no significant changes were observed in the weight and welfare of mice at the tested range concentrations. Only in the group receiving ISC with 5-FU was a slight decrease in body weight observed; however, this did not exceed 6% and was observed a short time after 5-FU injection (D9 and D23). An examination of organ mass showed that smaller spleens and smaller lung weights were observed in mice receiving 5-FU and the combination of ISC and 5-FU (5-FU effect) in comparison to the control. This is consistent with a lower number of leukocytes and number of metastases in the lung in groups receiving 5-FU.

## 3. Discussion

Antimetabolites (e.g., 5-FU) are one of the oldest groups of chemotherapy drugs used to treat solid tumors. 5-FU is commonly applied in combination with drugs from other classes of cancer chemotherapy agents, including alkylating agents, plant alkaloids, antimetabolites, anthracyclines, and topoisomerase inhibitors [3]. Due to the toxicity of those combined treatments and their insufficient anticancer activity, a search for new therapeutic options is still ongoing. For instance, 5-FU was combined with natural compounds, e.g., sulforaphane and quercetin, to modify its metabolism or cytotoxicity [11,15,16].

The anticancer effect of the combined treatment of ISC (organofluorine isoselenocyanate analogue of sulforaphane) and 5-FU has not yet been studied. In this study, their combination was examined in in vitro and in vivo models of TNBC.

The results of the in vitro study showed the synergic anticancer activity of the combined treatment in MDA-MB-231 cells. The effect of the combination of 5-FU and ISC was more potent than the effect of using both compounds alone and, in consequence, led to a drop in the effective concentration after applying compounds in combination in comparison to alone treatment. In cases of the most effective combined treatments, we observed a 10- or 20-fold (DRI = 10 or 20) reduction in the 5-FU dose. In clinical cases, reducing the dose of 5-FU is crucial to the success of chemotherapy due to the reduction in the drug’s side effects, which often limit therapy [16].

The mechanistic study revealed that the synergy of anticancer action resulted in the induction of apoptosis and necrosis. After the combined treatment, we observed fewer cells in the population; further, the microscopic study showed that combined treatment more strongly induced cell death than alone treatments with 5-FU or ISC. Commonly, apoptosis is still considered to be the main model of cell death in response to anticancer agents. Antimetabolites (e.g., 5-FU, gemcitabine) and their combined treatments with other drugs or natural compounds induce apoptosis (e.g., sulforaphane, apigenin, bufalin) [17,18,19,20]. When apoptosis is aborted, some clinically applied drugs can induce alternative cell deaths, e.g., necrosis (e.g., cisplatin) or autophagic cell death (e.g., tamoxifen) [21,22]. Of the two, necrosis is the least desirable because it triggers inflammation. The inflammation promotes cancer development by eliciting mitogenic or pro-survival cytokines production. Additionally, the inflammatory response damages normal tissue. On the other hand, the inflammation provokes the recruitment of cytotoxic immune cells to the tumor site and increases the effectiveness of chemotherapy [23,24]. Some studies have explored necroptosis (i.e., programmed necrosis) in order to induce cell death in drug-resistant breast cancer cells [25,26,27,28]. Both apoptosis and necrosis as a mechanism of increasing the effectiveness of anticancer drugs after their combination with natural compounds were demonstrated after combined treatment, e.g., diosmin with 5-FU in colorectal cancer cells and berberine with cisplatin in ovarian cancer cells [29,30].

The observed promising properties in in vitro conditions were verified in the mammary gland carcinoma 4T1 (TNBC) animal model. The experiments showed that the tumor growth inhibition was a result of additive interactions of combination components: 5-FU and ISC. Simultaneously, 5-FU alone treatment and combined treatment decreased the number of metastases by 50%, suggesting a dominating 5-FU effect. 5-FU is known to exhibit anti-metastatic efficacy in the 4T1 TNBC animal model [31]. Interestingly, we have shown in an in vitro model that this combined treatment permanently inhibits MDA-MD-231 cell migration, albeit in a similar way to after ISC alone treatment. Differences between in vivo and in vitro tests can be the result of another metabolism or other factor presented in the mouse organism.

At the last stage of study, the safety of the treatments was evaluated. All tested types of treatment were shown to be safe in vivo, despite the observation of some changes. First, we observed depletion of the spleen weight. VanderVeen et al., observed that during treatment with 5-FU, the weight of the spleen decreased and, consequently, the number of circulating leukocytes decreased [32]. The changes in the number of leukocytes were also revealed in our study after 5-FU alone treatment and after combined treatment, as well as in both cases at the same level, indicating that this is the effect of 5-FU. Another observed alteration after the combined treatment was a drop in lung weight, which was similar in the 5-FU group and combined treatment group. This effect of 5-FU was described earlier by Hashemzehi et al., after losartan and 5-FU combined treatment [33]. This phenomenon is often seen with drugs that reduce lung metastasis because reducing the number of the metastatic foci in the lungs reduces the weight of the lungs [31].

Our animal studies showed that the number of thrombocytes was elevated after combined treatment with ISC and 5-FU. In patients with breast cancer, an increase in the number of thrombocytes is disadvantageous because it is connected with poor prognosis and exerting an influence on apoptosis resistance, as well as the promotion of metastasis and the invasion of cancer cells [34,35,36]. Our studies showed that, in this case, the level of platelets was not the pivotal factor associated with metastasis. Despite a significant increase in the thrombocytes level after combined treatment in comparison to 5-FU or ISC alone treatments, the number of metastases in the lung was at the same level as after the 5-FU alone treatment.

In conclusion, our results revealed that ISC positively modulates the anticancer activity of common antimetabolite 5-FU. The combined treatment can be regarded as a promising anticancer strategy for highly aggressive and invasive triple-negative breast cancer treatment. ISC and 5-FU act synergistically in the in vitro model, and an additive type of interaction was observed in the in vivo model. Importantly, this combination was shown to be non-toxic in animals.

Bringing together the results obtained herein as well as the results of previous works, the distinct and promising potential of ISCs and other isothiocyanates to modify the action of antimetabolites is seen. In our opinion, further study on the combination treatment of ISCs with various antimetabolites in a triple-negative breast cancer model should be conducted in parallel with advanced mechanism-of-action research to fully understand the role of isothiocyanates in modulating the action of antimetabolites.

## 4. Materials and Methods

### 4.1. Cells and Reagents

The breast cancer cell lines MDA-MB-231 and 4T1 were obtained from the American Type Culture Collection (ATCC, Manassas, VA, USA). Cells were grown in IMDM (MDA-MB-231, Cytogen, GmbH Bienenweg, Berlin, Germany) or RPMI 1640 w/Gluta Max (4T1, Gibco, Grand Island, NY, USA). Mediums were supplemented with 10% fetal bovine serum (Gibco, Grand Island, NY, USA), 1% antibiotics solution (10,000 U/mL of penicillin and 10 mg/mL of streptomycin, 25 µg/mL of amphotericin B (only for MDA-MB-231 cells) (Sigma Aldrich, St. Louis, MO, USA)), and 1% nonessential amino acids (only for MDA-MB-231, Sigma Aldrich). To detect mycoplasma contamination, MycoAlert Assay Control Set, (Lonza, Verviers, Belgium) was used. 5-FU was obtained from Sigma Aldrich. ISC was synthesized by the Centre of Molecular and Macromolecular Studies Polish Academy of Sciences, Division of Organic Chemistry [13].

### 4.2. Cell Growth Assay

Cell growth was determined using the MTT (3-(4,5 dimethylthiazol-2-yl)-2,5 diphenyltetrazolium bromide) assay. Cells in the logarithmic phase of growth were used. When the cells reached 75% confluence, they were treated with increasing concentrations of compounds or their combination. A sequential scheme of experiments was applied, meaning that the cells were incubated for 24 h with ISC, followed by 72 h treatment with 5-FU. In the same experiments, the cells were treated with each of the compounds alone at concentrations corresponding to the concentrations tested in the combined treatment. At the end of the experiment, the medium was removed and the 0.25 mg/mL MTT (Sigma Aldrich) inserted. The absorbance was measured using the microplate scanning spectrophotometer (PowerWave X, BioTek, Winooski, VT, USA) at a wavelength of 570 nm with background subtraction at 690 nm.

### 4.3. Quantitative Analysis of Interactions

The Chou–Talalay method was used to quantitatively analyze the interactions. We tested concentrations that represented the multiplicity IC50 of the compounds. The quantification was based on the results of the MTT assay. To determine the type of interaction, the Combination Index (CI) was quantified:

CI = D1_comb_./D1_alone_ + D2_comb_./D2_alone_ (1)

D1_comb_. or D2_comb_.—concentrations of compound 1 or 2 that exert the certain effect in the combined treatment.

D1_alone_ or D2_alone_—concentrations of compound 1 or 2 used in the alone treatment that exert the same effect as in the combined treatment.

DRI = D_comb_./D_alone_ (2)

D_comb_.—concentration of compound used in the combined treatment.

D_alone_—concentration of compound used in the combined treatment.

Both concentrations have the same effect.

The value of the CI, CI > 1.1, CI < 0.9, and CI = 0.9 ÷ 1.1 indicates antagonism, synergism, and additive effects, respectively. CI and DRI were calculated using CompuSyn software (ComboSyn, Paramus, NJ, USA).

### 4.4. Identification of Apoptosis and Necrosis

For microscopy analyses, the cells were stained with the FITC Annexin V Apoptosis Detection Kit I (BD Biosciences Company, San Jose, CA, USA). An amount of 5 μL/mL of FITC Annexin V and 5 μL/mL PI were added on each well. The fluorescence was excited with 488 nm and PI 543 nm lasers, respectively, and was collected with 520 nm and 600 nm filters, respectively, using a confocal microscope (Olympus, Shinjuk, Tokyo, Japan) Living cells (AnnV−/PI−), early apoptotic cells (AnnV+/PI−), late apoptotic cells (AnnV+/PI+), and necrotic cells (AnnV−/PI+) were distinguished. The study was performed after 24 h of incubation. The assessment included sequential treatments with 2.75 μM of ISC and 38.6 μM of 5-FU, and the administration of compounds alone at concentrations equal to those used in the combination treatment.

### 4.5. Wound Assay

Cells were seeded in 8-well Nunc plates at a density of 2 × 10^5^ cells/mL. After 48 h, wounds were made by scraping across the confluent cell monolayer with a plastic 200 µL micropipette tip and rinsed several times with media. The cells were incubated in media with 5-FU, ISC, and their combination. Cells that migrated into the wound surface were determined under the microscope at various time points (0, 24, 48 h). The assessment included sequential treatments with 2.75 μM of ISC and 38.6 μM of 5-FU, and the administration of compounds alone at concentrations equal to those used in the combination treatment. The results were visualized by an inverted confocal microscope (Olympus IX70, Shinjuk, Tokyo, Japan) using differential interference contrast (DIC)/(Nomarski interference contrast) to better visualize unstained cells.

### 4.6. Animals

Female BALB/c mice were purchased from the Experimental Medicine Centre, Medical University of Bialystok, Poland. At 5–6 weeks of age, the animals were housed 3–4 per cage in an air-conditioned room at a temperature of 21–23 °C and humidity of 50%. All animal procedures were carried out in accordance with the Directive of The European Parliament on the protection of animals used for scientific purposes (2010/63/EU of European Parliament) and were approved by the Local Ethics Committee for Animal Experiments of the Ludwik Hirszfeld Institute of Immunology and Experimental Therapy PAS in Wrocław, 12 Weigla St., 53-114 Wrocław. Number of permits for animal experiments 34/2019.

### 4.7. Tumor Growth

Mice were injected *orthotopically* with 2 × 10^5^ of 4T1 murine breast cancer cells in 50 mL of PBS saline. When tumors were palpable (mean size reached 40 mm^3^), the mice were randomly divided into four groups *(n* = 8): control group, 5-FU alone treatment group, ISC alone treatment group, and combined treatment group. 5-FU (100 mg/kg m.c.) was administered intravenously (1× a week; Day 9, Day 23). ISC (50 mg/kg m.c.) was administered intraperitoneally (1× week; Day 9, Day 16, Day 23). Mice were sacrificed on Day 28. During autopsy, the heart, liver, lungs, and spleen were weighed.

Tumor size and animal weight were measured three times a week. Based on tumor size measurements (caliper, Mitutoyo Corp., CD-15DCX, Kanagawa, Japan), tumor volume (TV) and tumor growth inhibition (TGI%) were calculated:

TV = a^2^b/2 (3)

a—shorter diameter; b—longer diameter.

TGI = 100 − TV1/TV2100(4)

TV1—average tumor volume of treated mice.

TV2—average tumor volume of untreated control mice.

### 4.8. Determination Type of Interaction

The type of interaction between ISC and 5-FU indicated a comparable value of experimental TGI and the value of the calculated hypothetical tumor growth inhibition HTGI [4].

HTGI (%) = 100 − ((100 − TGI_5-FU_) × (100 − TGI_ISC_))/100 (5)

TGI_5-FU_—experimental TGI for 5-FU alone.

TGI_ISC_—experimental TGI for ISC alone.

A synergistic interaction was observed when the experimental TGI was greater than HTGI. Antagonism was noticed when the experimental TGI was smaller than HTGI. An additive effect was found when the value of the experimental TGI and HTGI were comparable.

### 4.9. Blood Analysis

Blood samples were collected in 1 mL vials with EDTA2KEDTA2K, and a blood morphological analysis was performed using the Mythic18 (Orphee) hematology analyzer. Then, the blood was centrifuged (2400× *g*, 12 min, 4 °C) into plasma isolation and stored at −80 °C. A biochemical analysis was performed on the plasma using the Cobas c111 biochemistry analyzer (Roche, Basilea, Switzerland), and the following parameters were determined: ALT, AST, creatinine, urea, creatine kinase (CK), and CK-MB isoenzyme.

### 4.10. Metastatic Foci Quantification

The lungs were fixed in 4% paraformaldehyde. A blinded macroscopic count for metastatic foci on the surface of lung tissue was performed [37]. Only visible macroscopic metastases in the lungs were counted.

### 4.11. Statistical Analysis

Data are presented as the mean value ± standard deviation (S.D.). *p* < 0.05 was considered statistically significant.

The results of the MTT assay and blood analyses were carried out using a one-way analysis of variance (ANOVA), followed by a post hoc Tukey’s test, which was used to compare pairs of group means. The analyses were performed with GraphPad Prism 7 (GraphPad Software, Inc., La Jolla, CA, USA).

The results of in vivo studies (expect blood analysis) were analyzed by employing STATISTICA version 10 (StatSoft, Inc., Tulsa, OK, USA) using the Mann–Whitney U test.

## Figures and Tables

**Table 1:** Experimental and theoretically calculated tumor growth inhibition (TGI, %).

Day	TGI	Hypothetical TGI
13	59.8	63.9
15	55.0	59.3
17	56.8	61.3
20	51.9	51.1
22	51.6	55.3
24	50.9	49.5*
27	50.5	52.2

* Synergistic effect of ISC and 5-FU (experimental TGI exceeds theoretically calculated TGI).

**Table 2:** Blood count.

		Group				
	Unit	Healthy Mice	Control	5-FU	ISC	ISC + 5-FU
Leukocytes	[10^3^/μL]	5.9 ± 1.5 *	216 ± 95	3.4 ± 1.6 *	208 ± 89	4.9 ± 2.8 *^,†^
Lymphocytes	%	80.3 ± 5.4 *	14.4 ± 3.6	85 ± 6.4 *	17.3 ± 4.3	77.3 ± 8.1 *^,†^
Monocytes	%	4.4 ± 1 *	7.5 ± 0.6	2.7 ± 1 *	10.4 ± 0.9 *	2.1 ± 0.4 *^,†^
Granulocytes	%	15.3 ± 6 *	78.2 ± 4	12.2 ± 5.9 *	72.3 ± 4.9	20.6 ± 7.8 *^,†^
Erythrocytes	[10^6^/μL]	8.1 ± 1.4	8.7 ± 0.6	6.9 ± 1 *	8.8 ± 0.5	6.3 ± 1 *^,†^
Hemoglobin	[g/dL]	13.8 ± 0.7 *	18.6 ± 1.3	14.2 ± 2.7 *	18.6 ± 1.1	12.8 ± 1.8 *^,†^
Hematocrit	%	39.2 ± 1.68 *	46.4 ± 2.5	33.5 ± 5 *	47.4 ± 3.5	30.4 ± 4.2 *^,†^
MCV	[fL]	48.3 ± 0.9 *	53.3 ± 2.6	48.5 ± 0.7 *	54 ± 1.6	48.7 ± 4.2 *^,†^
Platelets	[10^3^/μL]	482.0 ± 26.8	680.0 ± 123.0	971.0 ± 243.0	770.0 ± 97.0	1512 ± 356 *^,†,‡^

*—Tukey’s test vs. control *p* < 0.05, ^†^—Tuckey test vs. ISC alone treatment *p* < 0.05, ^‡^—Tuckey test vs. 5-FU alone treatment *p* < 0.05.

**Table 3:** Biochemical parameters of mice blood.

		Group			
	Unit	Control	5-FU	ISC	ISC + 5-FU
ALT	U/L	27.0 ± 11.5	17.4 ± 3.2	23.2 ± 6	17.6 ± 2.9
AST	U/L	159.5 ± 55.1	95.5 ± 27.6	154.9 ± 66.9	80.9 ± 15.9 ^†,^*
Creatinine	μmol/L	6.5 ± 1.8	5.6 ± 2.9	6.5 ± 1.1	6.4 ± 2.1
Urea	mmol/L	6.0 ± 0.5	6.7 ± 1.4	5.9 ± 1	6.4 ± 1.4
Creatinine kinase [CK]	U/L	1137.7 ± 772.3	983.6 ± 448.6	1428.2 ± 1018.3	785.3 ± 248.5
CK-MB [myocardial CK]	U/L	394.0 ± 171.9	271.6 ± 60.5	378.2 ± 109.8	234.5 ± 52.7 *

* Tuckey test vs. control *p* < 0.05, ^†^ Tuckey test vs. ISC alone treatment *p* < 0.05.

## Data Availability

Data is contained within this article.
